# A dysfunctional endolysosomal pathway common to two sub-types of demyelinating Charcot–Marie–Tooth disease

**DOI:** 10.1186/s40478-020-01043-z

**Published:** 2020-10-15

**Authors:** James R. Edgar, Anita K. Ho, Matilde Laurá, Rita Horvath, Mary M. Reilly, J. Paul Luzio, Rhys C. Roberts

**Affiliations:** 1 Cambridge Institute for Medical Research, University of Cambridge, Cambridge Biomedical Campus, Cambridge, CB2 0XY UK; 2 Present Address: Department of Pathology, University of Cambridge, Tennis Court Road, Cambridge, CB2 1QP UK; 3 Department of Biology, University of York, York, YO10 5DD UK; 4 Centre for Neuromuscular Diseases, UCL, Queen Square Institute of Neurology, University College London, London, WC1N 3BG UK; 5 Department of Clinical Neurosciences, University of Cambridge, Cambridge, CB2 0PY UK

**Keywords:** Charcot–Marie–Tooth, Peripheral neuropathy, LITAF, Endosome, Lysosome, Phosphoinositide

## Abstract

Autosomal dominant mutations in *LITAF* are responsible for the rare demyelinating peripheral neuropathy, Charcot–Marie–Tooth disease type 1C (CMT1C). The LITAF protein is expressed in many human cell types and we have investigated the consequences of two different *LITAF* mutations in primary fibroblasts from CMT1C patients using confocal and electron microscopy. We observed the appearance of vacuolation/enlargement of late endocytic compartments (late endosomes and lysosomes). This vacuolation was also observed after knocking out LITAF from either control human fibroblasts or from the CMT1C patient-derived cells, consistent with it being the result of loss-of-function mutations in the CMT1C fibroblasts. The vacuolation was similar to that previously observed in fibroblasts from CMT4J patients, which have autosomal recessive mutations in *FIG4*. The FIG4 protein is a component of a phosphoinositide kinase complex that synthesises phosphatidylinositol 3,5-bisphosphate on the limiting membrane of late endosomes. Phosphatidylinositol 3,5-bisphosphate activates the release of lysosomal Ca^2+^ through the cation channel TRPML1, which is required to maintain the homeostasis of endosomes and lysosomes in mammalian cells. We observed that a small molecule activator of TRPML1, ML-SA1, was able to rescue the vacuolation phenotype of LITAF knockout, FIG4 knockout and CMT1C patient fibroblasts. Our data describe the first cellular phenotype common to two different subtypes of demyelinating CMT and are consistent with LITAF and FIG4 functioning on a common endolysosomal pathway that is required to maintain the homeostasis of late endosomes and lysosomes. Although our experiments were on human fibroblasts, they have implications for our understanding of the molecular pathogenesis and approaches to therapy in two subtypes of demyelinating Charcot–Marie–Tooth disease.

## Introduction

The Charcot–Marie–Tooth diseases (CMT) encompass a large group of progressive inherited peripheral neuropathies leading to muscle atrophy, weakness and sensory impairment of the limbs. Thanks to advances in DNA sequencing, more than 100 genes are now known to be associated with CMT [19, 34, 41]. Subtypes of CMT have long been classified into ‘axonal’ and ‘demyelinating’ subgroups using neurophysiological criteria. This distinction has proved helpful by reflecting the presumed primary sites of underlying cellular dysfunction as the axons of peripheral nerves or the supporting Schwann cells, respectively. Schwann cells, derived from the neural crest, engulf peripheral nerve axons during development and, when triggered to do so via the stimuation of surface receptors by specific growth factors, wrap layers of plasma membrane around larger axons to form myelin. This in turn leads to the clustering of ion channels in highly specialised regions called nodes of Ranvier, facilitating the efficient propagation of action potentials along axons from the spinal cord to the peripheries and vice versa. ‘Demyelinating’ subtypes of CMT are therefore characterised by the defective formation or maintenance of the myelinated state by the Schwann cell, eventually leading to axonal degeneration and clinical manifestation [3].

Of the genes known to be associated with demyelinating CMT, three main functional categories emerge: genes encoding proteins that play a structural role in the formation and maintenance of the myelin sheath; genes encoding transcription factors that are crucial in activating the Schwann cell’s myelination programme when appropriately stimulated; and, finally, genes encoding proteins known or predicted to function in intracellular membrane traffic. In the latter group, most of these proteins are widely expressed across different tissues and cell types, and several are known to function specifically in the endolysosomal pathway [5]. This suggests the existence of pathogenic mechanisms—and hence potential therapeutic targets – that are common to more than one subtype of CMT. For example, mutations in *FIG4* and *MTMR2/13* are associated with the autosomal recessive subtypes of demyelinating CMT known as CMT4J [6, 30, 44] and CMT4B [17], respectively. These genes encode proteins involved in endolysosomal function; FIG4 is a lipid phosphatase which, in conjunction with the scaffolding protein, VAC14, and the lipid kinase, PIKfyve, forms part of the PIKfyve protein complex responsible for generating PtdIns(3,5)P2 (phosphatidylinositol 3,5-bisphosphate) from PtdIns(3)P (phosphatidylinositol 3-phosphate) at the limiting membrane of endosomes [9, 18]. In turn, PtdIns(3,5)P2 regulates the homeostasis of endosomes and lysosomes in mammalian cells by mechanisms including the activation of the lysosomal cation channel TRPML1 (transient receptor potential mucolipin 1) [2], resulting in the release of localised Ca2 + stores [8]. In contrast, MTMR2/13 exerts an effect on endolysosomes by dephosphorylating both PtdIns(3)P and PtdIns(3,5)P2 leading to a proposed functional link with FIG4 [17, 42].

CMT1C is an autosomal dominant form of demyelinating CMT associated with mutations in *LITAF* (Lipopolysacharide-induced tumour necrosis factor-α factor). *LITAF*, also known as *SIMPLE* (Small integral membrane protein of the lysosome/late endosome), encodes a widely-expressed 17 kDa monotopic integral membrane protein composed of an N-terminal proline-rich region and an evolutionary conserved zinc-binding C-terminal ‘LITAF domain’ that anchors the protein to endosomal membranes [14, 32]. Furthermore, a cargo sorting role in endocytic membrane trafficking is supported by the ability of the proline-rich region of LITAF to interact with the ESCRT protein, TSG101 [20, 37], as well as the ITCH/NEDD4 family of E3-ubiquitin ligases [10, 37]. We and others have shown that both the N- and C-termini of LITAF are present on the cytosolic face of endosomes and that membrane association is mediated by the C-terminal LITAF domain via a predicted hydrophobic helix and specific interactions with phosphoethanolamine (PE) head groups [14, 32]. Interestingly, all CMT1C-associated pathogenic mutations are found in and around this hydrophobic region within the C-terminal LITAF domain and have been proposed to disrupt membrane interaction and lead to protein instability [14, 21].

Despite our expanding knowledge concerning the genes associated with CMT, no reversible treatments currently exist. The development of targeted effective treatments for CMT will therefore rely on improving our understanding of the underlying cellular pathomechanisms for each subtype. Moreover, some individual subtypes of CMT linked to single genes might in fact require different treatment approaches depending on the specific mutation, thereby adding an additional level of complexity to drug development. While therapeutic strategies aimed at introducing functional wild type copies of CMT-asscociated genes encoding proteins involved in membrane trafficking into Schwann cells have shown real promise in autosomal recessive forms of the disease using viral delivery systems [35], the optimal therapeutic approach towards autosomal dominant subtypes such as CMT1C remains unclear. A key question that needs answering before treatments for CMT1C can be developed is whether the expression of a single copy of the *LITAF* gene harbouring disease-associated mutations causes a toxic ‘gain of function’, or whether the heterozygous presence of the mutated protein leads to loss of function in either a ‘haploinsufficent’ or ‘dominant-negative’ fashion, mimicking any phenotype that might be the result of the absence of wild-type protein expression in vivo.

Given the localisation of LITAF to endosomes and the prediction that disease-causing mutations disrupt the interaction with membranes and lead to protein instability [14, 21], we set out to determine the consequences of these mutations on the morphology of endosomal and lysosomal compartments in primary fibroblasts from two CMT1C patients harbouring different pathogenic *LITAF* mutations. Having established the cellular phenotype, we then compared the observed morphology with that of the same compartments in fibroblasts depleted of LITAF or the CMT4J-associated protein, FIG4. Finally, we investigated the rescue of these observed phenotypes with a small molecule activator of TRPML1, previously shown to rescue the phenotype associated with mutations in *FIG4* [46], allowing us to propose a potential therapeutic target common to more than one subtype of CMT.

## Materials and methods

### Antibodies

Antibodies used for immunofluorescence were as follows; anti-LAMP1; mouse monoclonal, H4A3 from Santa-Cruz, anti-Cathepsin D; rabbit polyclonal, 219361 from Calbiochem, anti-APPL1; rabbit polyclonal, H-96 from Santa Cruz, anti-EEA1; mouse monoclonal, 610457 from BD Transduction, anti-CD63; mouse monoclonal, IB5, a gift from Prof. Mark Marsh (University College London), anti-LBPA; mouse monoclonal, 6C4, a gift from Prof. Jean Gruenberg (University of Geneva), anti-ubiquitin; mouse monoclonal, FK2 from Enzo Life Sciences, anti-LC3B; rabbit polyclonal, 2775S from Cell Signalling Technology, anti-CIMPR; mouse monoclonal, ab2733 (2G11) from Abcam, anti-TGN46; sheep polyclonal, AHP500 from Bio-Rad, anti-LITAF; mouse monoclonal, 611614 from BD Biosciences.

Antibodies used only for Western blotting were anti-FIG4; rabbit polyclonal, ab221758 from Abcam, anti-alpha-Tubulin; mouse monoclonal, T9026 from Sigma.

### Fibroblast generation

Human skin fibroblasts were collected by biopsy from 2 patients with CMT1C via the Centre for Neuromuscular Diseases, UCL and at Newcastle University. Fibroblasts were supplied by the MRC Centre for Neuromuscular Disease Biobank London (REC reference number 06/Q0406/33) for one patient. An age matched control to the fibroblasts harbouring mutations (ages 39 and 46) was also collected. All fibroblast cultures were grown in high-glucose DMEM supplemented with 10% FBS, 2 mM Glutamax and 50 U/ml penicillin and 50 µg/ml streptomycin. Patients gave consent to the studies “Genotype and phenotype in inherited neurological diseases” study (REC: 13/YH/0310, IRAS ID: 2042290) and CMT: A Natural History Study (IRAS ID 30165; REC: 09/H0716/61).

### Lentivirus generation and transduction

Mixed knockout fibroblasts were generated using a lentiviral CRISPR/Cas9 method. Lentiviral vectors (see below) were cotransfected into HEK293ET cells with pCMVR8.91 (“199”) and pMD.G (VSV-G) (“200”) packaging plasmids using TransIT-293 (Mirus Bio, USA). Viral supernatants were collected 48 h after transfection, passed through 0.45 μm filters and fibroblasts transduced by ‘spinfection’—viral supernatants were centrifuged at 1800 rpm in a benchtop centrifugue at 37 °C for 3 h to enhance viral transduction.

Fibroblasts were first transduced with Cas9-encoding lentivirus generated using pHRSIN-SFFV-Cas9-PGK-Hygro. 48 h after transduction, cells were selected with 200 µg/ml hygromycin for 9 days. GuideRNAs were cloned into the lentiviral pKLV-U6gRNA-EF-(BbsI)-PGK-Puro2A-BFP vector, and 48 h after transduction cells were selected with 1 µg/ml puromycin for 5 days.

Guide RNA sequences; LITAF (5′ CACCGACAACACATTTGGATAGGG 3′), FIG4 (5′ CACCGGAGTTTTCCATCCCACTG 3′).

Polyclonal fibroblasts were generated and assayed for expression of the LITAF/FIG4 protein by immunofluorescence and Western blotting.

### Immunofluorescence

Cells were grown on glass coverslips and fixed using 4% PFA/PBS. Cells were quenched with 15 mM glycine/PBS and permeabilised with 0.1% saponin/PBS. Blocking and subsequent steps were performed with 1% BSA, 0.01% saponin in PBS. Cells were mounted on slides with mounting medium containing DAPI (Invitrogen). Cells were imaged using a LSM780 confocal microscope (63×/1.4 NA oil immersion objective; ZEISS).

### Conventional electron microscopy

Unless stated otherwise, cells were fixed with 2% PFA, 2.5% glutaraldehyde, 0.1 M cacodylate buffer, pH 7.2 before being scraped and pelleted. Pellets were secondarily fixed with 1% osmium tetroxide/1.5% potassium ferrocyanide and then incubated with 1% tannic acid in 0.1 M cacodylate buffer to enhance membrane contrast. Cells were dehydrated using increasing percentages of ethanol before resin was infiltrated using a 1:1 mix of propylene oxide and CY212 Epoxy resin. Samples were then transferred into neat CY212 Epoxy resin before being embedded in beam capsules. Resin was cured overnight at 65 °C. Ultrathin sections were cut using a diamond knife mounted to a Reichart ultracut S ultramicrotome. Sections were collected onto piloform-coated slot grids and stained using lead citrate. Sections were viewed on a FEI Tecnai transmission electron microscope at a working voltage of 80 kV.

### Cryo-immunogold electron microscopy

Cells were fixed with 4% PFA/0.1% glutaraldehyde/0.25 M HEPES buffer pH7.4, scraped and pelleted. Pellets were supported in 10% gelatine before being infused with sucrose/PVP. 70 nm sections were cut at − 120 °C using a Diatome diamond knife mounted to a Leica UC7 cryo ultramicrotome. The sections were picked up with either 2.3 M sucrose, or a mixture of methylcellulose and sucrose and placed onto formvar and carbon-coated gold grids.

Thawed sections were subsequently labelled using antibodies followed by protein-A gold as previously described [39]. Murine monoclonal antibodies were followed by bridging using rabbit anti-mouse antibodies, before being labelled with protein-A gold. Sections were visualised on a FEI Tecnai transmission electron microscope at a working voltage of 80 kV.

### BSA-gold uptake

BSA-gold was prepared as previously described [38]. Cells were grown on Thermanox coverslips and incubated with BSA-gold continuously for 2 h before being washed with PBS, fixed and prepared for conventional EM as above.

### HRP-DAB chemistry

Cells were grown on Thermanox coverslips and incubated with 5 mg/ml HRP in culture media for 4 h, before being washed four times with PBS and chased in full media overnight. Cells were then fixed with 2% PFA, 2.5% glutaraldehyde, 0.1 M cacodylate buffer before being washed with 15 mM glycine. Subsequently, cells were incubated with 1.5 mg/ml DAB (3, 3,’-Diaminobenzidine), in TBS (pH 7.6) containing 0.05% H_2_O_2_ at 4 °C, in the dark for 30 min. Cells were washed with 0.1 M cacodylate buffer before being post-fixed with 1% osmium:1.5% potassium ferrocyanide, and subsequently processed as described above.

### Correlative Light and Electron Microscopy (CLEM)

Fibroblasts were plated to gridded coverslips with alpha-numeric coordinates (P35G-1.5-14-CGRD, MatTek) and left to settle before being transfected with 2 μg PX-GFP plasmid using Xtreme Gene HP (Roche). Two days following transfection, cells were fixed with 2% PFA, 2.5% glutaraldehyde, 0.1 M cacodylate buffer. Cells were then stained with Hoechst (to stain the nucleus) for 2 min, before being washed with 0.1 M cacodylate buffer. PX-GFP fluorescence was imaged on an LSM780 confocal microscope (63×/1.4 NA oil immersion objective; ZEISS) and the coordinates of cells selected for imaging were recorded. To prepare for electron microscopy, cells were secondarily fixed with 1% osmium tetroxide/1.5% potassium ferrocyanide and then incubated with 1% tannic acid in 0.1 M cacodylate buffer to enhance membrane contrast. Samples were then processed as previously described until embedding, where pre-baked epoxy stubs were inverted over areas of interest. After baking, the dish was removed using liquid nitrogen. Areas of interest were identified by alpha-numeric coordinates and 70 nm ultrathin sections were collected using a Diatome diamond knife attached to an ultracut UCT ultramicrotome (Leica). Sections were stained with lead citrate before being imaged on a Tecnai Spirit transmission electron microscope (FEI) at an operating voltage of 80 kV.

### Western blots

Fibroblasts were rinsed once in PBS and lysed in RIPA buffer containing 50 mM Tris, pH 7.4, 150 mM sodium chloride, 1% NP-40, 0.5% sodium deoxycholate, 0.1% SDS, 1 mM EDTA, complete protease inhibitor (Roche). Lysates were centrifuged at 20,000×*g* at 4 °C for 20 min and the supernatant was retained. The protein concentration of the lysed sample was determined using Precision Red reagent (Cytoskeleton, Inc.). Equal amounts of protein were mixed with NuPAGE® LDS Sample Buffer (Thermo Scientific) and were heated at 70 °C for 10 min. Samples were separated by SDS–PAGE, followed by semi-dry transfer onto a PVDF membrane (Whatman). Membranes were blocked with 5% (w/v) dried milk in PBS prior to incubation with primary antibody. The membrane was washed with PBST (PBS, 0.3% Tween) first for 15 min, then twice for five minutes before incubation with Horseradish Peroxidase (HRP)-Conjugated secondary antibody. Western blots were developed using Pierce ECL Western Blotting Substrate (Thermo scientific).

### ML-SA1

The synthetic TRPML1 activator, ML-SA1 (Tocris Bioscience) was dissolved in DMSO at a stock concentration of 20 mM. ML-SA1 was used at a final concentration of 40 µM in full media for 36 h as previously described [46].

### Lysosome quantification

To determine the proportion of cells with enlarged late endosomes/lysosomes, fixed cells were permeabilised, labelled with LAMP1 and processed for immunofluorescence microscopy as previously described. At least 23 cells were recorded per cell line, per independent experimental conditions. Cells were imaged at random, and the largest LAMP1-positive organelle per cell was measured using ZEN Black (Zeiss). Three-independent experiments were performed, allowing means and standard error of means calculated.

Unpaired, two-tailed *T* tests were performed. *p* < 0.05 *, *p* < 0.01 **, *p* > 0.001 ***.

## Results

**Fig. 1:**
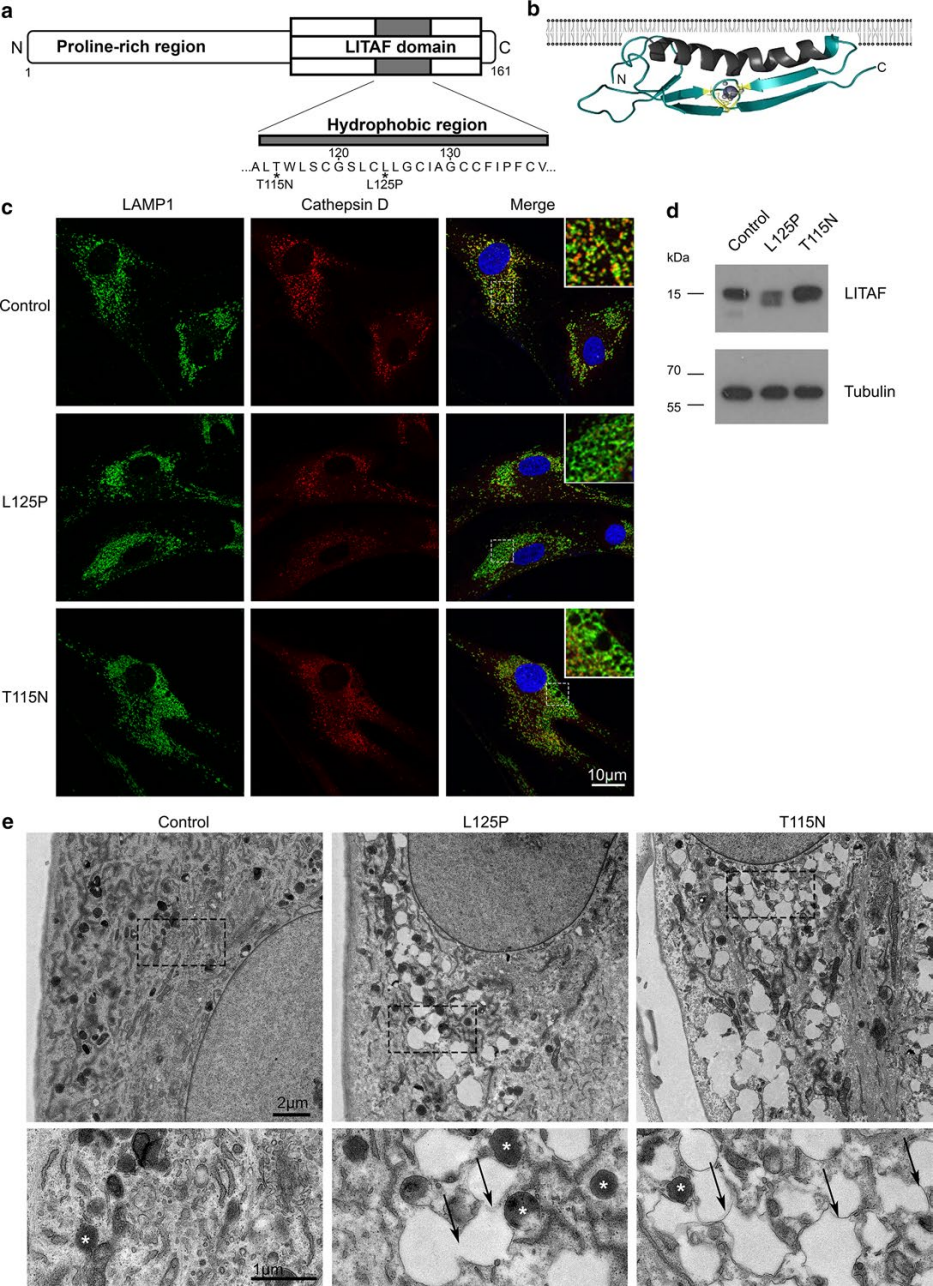
Patient-derived fibroblasts with mutations in LITAF display enlarged late endocytic compartments. **a** Schematic diagram to illustrate the domain organisation of the LITAF protein. The N terminus is characterised by a proline-rich region, followed by a ‘LITAF domain’ at the C terminus. Both T115N and L125P mutations are within the hydrophobic region of the LITAF domain (grey). **b** Diagram of the topology of the LITAF domain within the phospholipid bilayer (created with Adobe Photoshop). The predicted hydrophobic anchor (grey) of the LITAF domain, shown as a Rosetta structural model (from Ho et al. [14]), is embedded in the membrane's phospholipid bilayer, but does not traverse it. **c** Representative confocal immunofluorescence images of control fibroblasts, and CMT1C patient-derived fibroblasts with mutations in LITAF (L125P, T115N) display enlarged late endocytic compartments, as shown by LAMP1 (green) and Cathepsin D (red) staining. Insets show LAMP1 staining in ‘rings’ as opposed to fine puncti in LITAF mutant cells. **d** Protein lysates were analysed by immunoblotting with an antibody raised against LITAF to confirm the expression of LITAF from control and CMT1C patient-derived fibroblasts. Alpha-tubulin served as loading control. Full-length blots are shown in Additional file 2: Fig. 2e) Conventional electron microscopy of control and LITAF patient-derived fibroblasts reveal abnormal, swollen, vacuolar compartments in LITAF mutant cells (L125P, T115N). Insets reveal vacuoles to be membrane bound; black arrows show interconnections, and white asterisks show dense core lysosomes

We obtained fibroblasts from the skin of two CMT1C patients with known mutations in LITAF (L125P; p.Leu125Pro and T115N; p.Thr115Asn), and age- and sex-matched controls. The clinical features of the patients are summarised in Table 1. Both mutations are within the hydrophobic region of the LITAF domain and are predicted to disrupt its helical propensity and interaction with endosomal membranes [14] (Fig. 1a). LITAF domains are conserved in eukaryotes and target proteins to membranes, but do not traverse the lipid bilayer, rather becoming anchored to the membrane via an in-plane helical membrane anchor [14] (Fig. 1b). To determine whether the mutations in LITAF altered the number or position of late endosomes/lysosomes, we performed confocal immunofluorescence microscopy and observed that these organelles were enlarged in both patients’ fibroblasts, by staining with the late endosome/lysosome markers LAMP1 (lysosomal-associate membrane protein 1) and Cathepsin D (Fig. 1c). LAMP1 could routinely be observed as rings, rather than puncta in both L125P and T115N fibroblasts, and Cathepsin D staining often appeared to be lost from, or moved to the edge of, these enlarged structures. Interestingly, no other endomembrane-related organelle markers that we tested displayed consistent differences in staining patterns (Additional file 1: Fig. 1). There was no alteration in the staining patterns of the early endosome markers APPL1 (adaptor protein, phosphotyrosine interacting with PH domain and leucine zipper 1) and EEA1 (early endosome antigen 1), the trans-Golgi network marker TGN46 or the ciMPR (the cation independent mannose 6-phosphate receptor), which shuttles between the TGN and endosomes to deliver newly synthesised lysosomal hydrolases. Despite the alterations in LAMP1 and Cathepsin D staining, there was no obvious change in staining the late endosome/lysosome markers CD63 and LBPA (lysosbisphosphatidic acid), but these are both found mainly on intraluminal membranes in contrast to LAMP1 which labels mainly the limiting membrane of late endosomes and lysosomes [13, 43]. The lack of any increase in ubiquitin staining or of LC3 staining in the patients’ fibroblasts is consistent with no significant alteration in autophagy. By western blotting, we examined the levels of LITAF in the patients’ cells. No change in the level of LITAF was observed in the T115N patient fibroblasts compared to controls, but there was some reduction in the L125P cells, implying that this mutation may result in increased instability of the protein (Fig. 1d, Additional file 2: Fig. 2). Nevertheless there were easily detectable concentrations of LITAF in the fibroblasts from both patients.

**Table 1:** Clinical features

Patient	1	2
Mutations	c.374 T > C; p.Leu125Pro	c.334G > A p.Thr115Asn
Sex/age	M/39	F/46
Age of onset	First decade	30 years
Presenting symptom	High arches,	Foot deformity (asymmetric), pain on longer walks
Weakness		
Upper limbs	−	−
Lower limbs	+	+
Pinprick		
Upper limbs	−	−
Lower limbs	++	++
Vibration		
Upper limbs	−	−
Lower limbs	+	+
Skeletal deformities	Severe pes cavus	Pes cavus, hammer toes
CMTES	6	7
CMTNS	ND	9
Aid for walking	No	No

Weakness: − normal, +  ≥ 4 in distal muscles, ++ < 4 in distal muscles, +++ proximal weakness (knee flexion and extension, elbow flexion and extension, and/or above); Pinprick and vibration sense: − normal, + reduced below wrist/ankle, ++ reduced below elbow/knee, +++ reduced at or above elbow/knee

*N*  normal, *ND* not done, *VEP* visual evoked potentials, *BAEP*  brain stem auditory evoked potentials, *CMCT*  central motor conduction time, *CMTES*  Charcot–Marie–Tooth evaluation score (max score 28/28), *CMTNS*  Charcot–Marie–Tooth Neuropathy Score (max score 36/36)

**Fig. 2:**
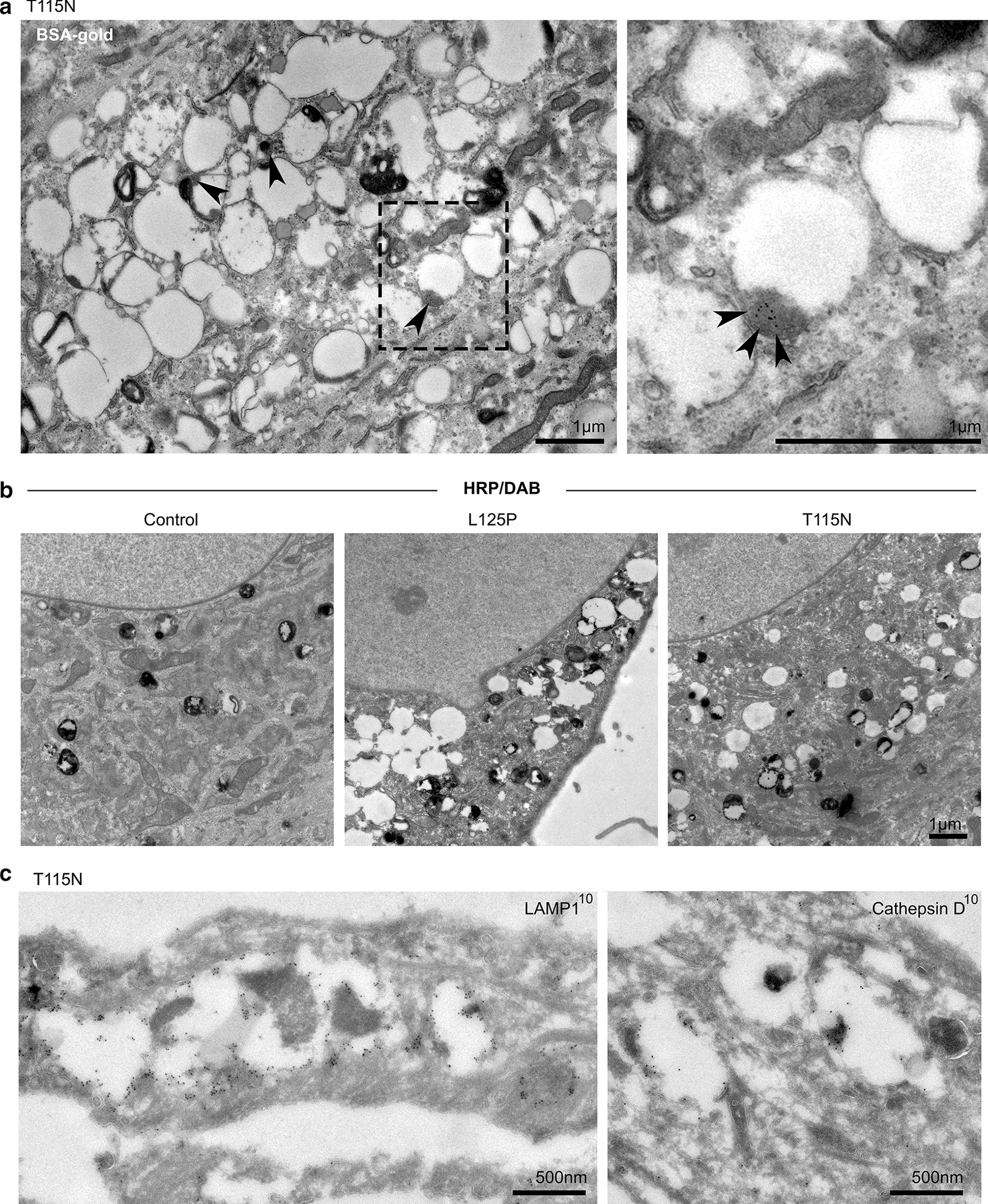
Characterisation of swollen compartments in LITAF mutant fibroblasts. **a** T115N patient-derived fibroblasts were incubated with the endocytic tracer BSA-gold for 16 h, and followed by a 4 h chase period to load lysosomes with BSA-gold. Conventional electron microscopy reveals the presence of flocculated BSA-gold within electron dense portions of vacuolated compartments (arrowheads). **b** Control or LITAF mutant fibroblasts were incubated with fluid-phase HRP for 4 h before being fixed. A DAB reaction was performed on cells before being processed for conventional electron microscopy (further examples in Additional file 3: Fig. 3). **c** Cryo immunogold electron microscopy was performed on T115N patient fibroblasts. Labelling with anti-LAMP1 antibody shows vacuolated compartments to be LAMP1 positive, and labelling with anti-Cathepsin D antibodies reveals labelling on electron dense patches within the lumen of such swollen compartments

**Fig. 3:**
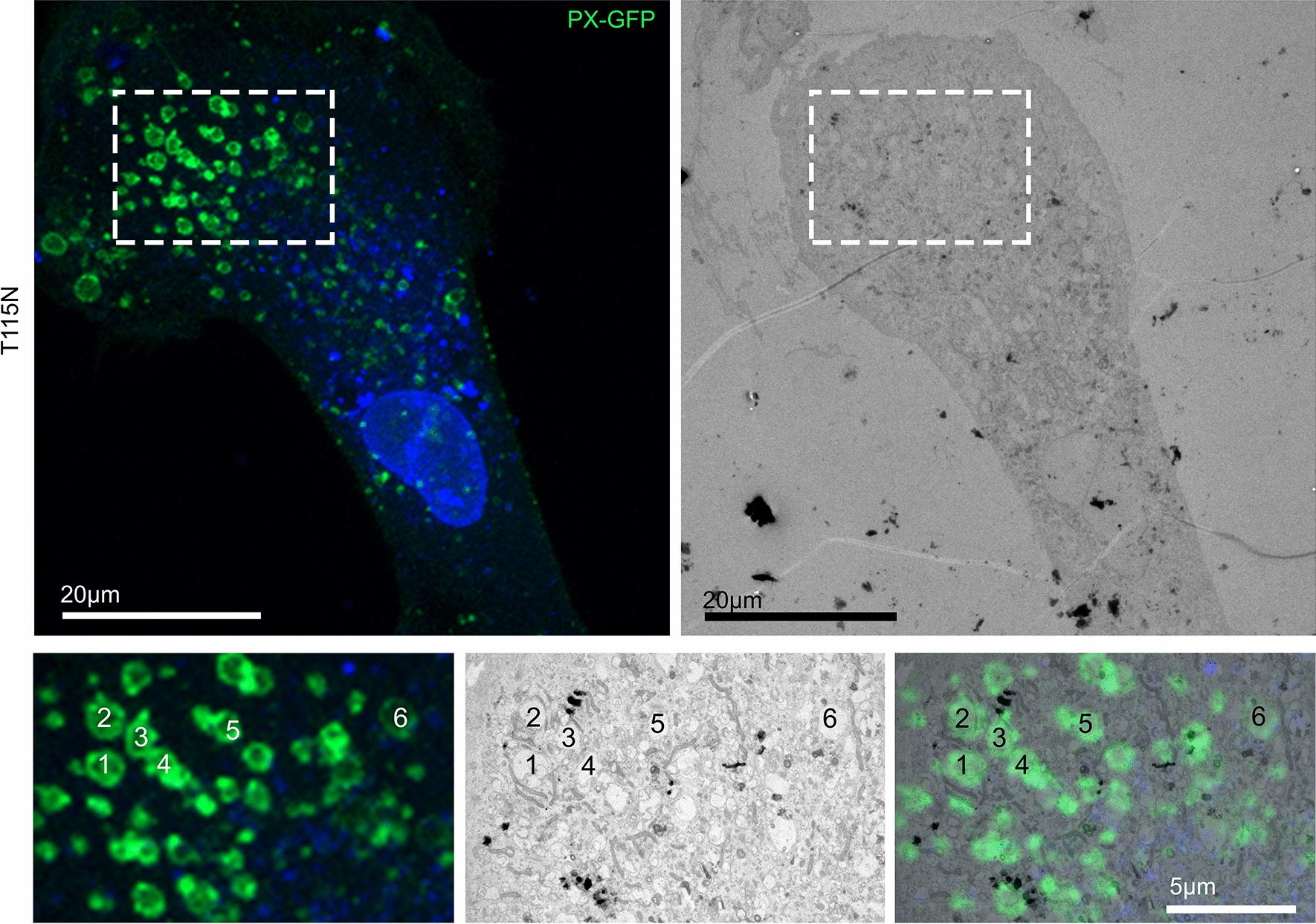
Correlative Light and Electron Microscopy of T115N patient fibroblasts identifies vacuolated compartments to be PtdIns(3)P positive. CLEM of T115N patient fibroblast transfected with the PtdIns(3)P-probe, PX-GFP. Insets display the overlap between PX-GFP fluorescence and appearance of vacuolar structures by electron microscopy

To investigate the phenotype of enlarged late endosomes and lysosomes further, we performed conventional transmission electron microscopy (EM) on control and patient-derived fibroblasts. The fibroblasts from both patients contained abnormal, large, vacuolated compartments, with the membranes of these swollen compartments often appearing to be interconnected (Fig. 1e). To clarify further the endocytic nature of these vacuolated compartments, we incubated T115N cells with endocytic tracers that are taken up by fluid phase endocytosis and can be visualised by EM. Both endocytosed BSA-gold and the electron dense product of horseradish peroxidase (HRP) catalysis were found within vacuolated organelles (Fig. 2a, b). BSA-gold was observed to be flocculated, indicating that the BSA had been hydrolysed [4]. The resultant gold particles were preferentially localised to the dense cores of the vacuolated organelles, highlighted by arrowheads (Fig. 2a). Cryo-immunogold EM of T115N fibroblasts confirmed that the vacuolated compartments were positive for the late endosome/lysosome markers—LAMP1 was localised to be limiting membrane and Cathepsin D to the dense cores within the lumen of the vacuolated structures (Fig. 2c, Additional file 3: Fig. 3). To assess the localisation of PtdIns(3)P, we performed CLEM (correlative light and electron microscopy) on T115N patient fibroblasts transfected with the PtdIns(3)P reporter PX-GFP. By fluorescence microscopy, the PX-GFP was found in small puncta consistent with its expected endosomal localisation, but also in larger compartments, which correlated with enlarged, vacuolated structures as seen by electron microscopy (Fig. 3).

**Fig. 4:**
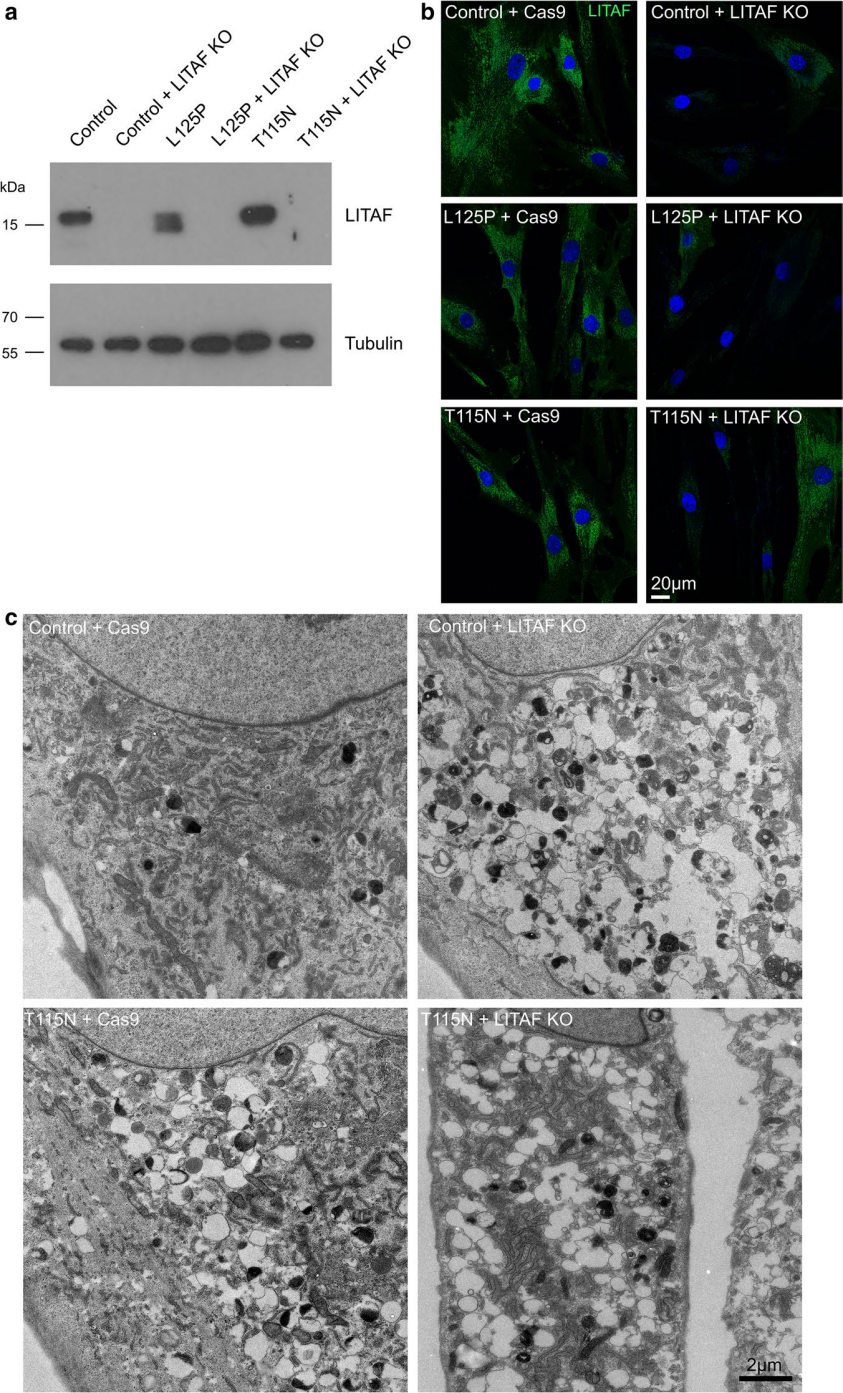
Mutations in LITAF result in loss-of-function, through a dominant-negative mechanism, rather than by toxic gain-of function. LITAF knockout (KO) Control, L125P and T115N patient fibroblasts were generated by CRISPR/Cas9. Antibiotic selection was used to maintain Cas9 and gRNA transduced cells, generating mixed populations of KO cells. **a** Western blotting was performed to confirm the loss of LITAF protein from mixed cell populations. Alpha-tubulin served as loading control. Full-length blots are shown in Additional file 2: Fig. 2. **b** Representative confocal microscopy images of fixed fibroblast lines that had been stained using an anti-LITAF antibody, to show the loss of LITAF expression from ~ 95% of cells. **c** Electron micrographs of control fibroblasts expressing Cas9, and following LITAF KO, and Cas9 expressing T115N fibroblasts, and following LITAF KO. LITAF KO cells display swollen endocytic compartments from either control backgrounds or T115N backgrounds

To determine whether mutations in LITAF were inducing the observed phenotypes, or whether mutations were inhibiting the function of LITAF (toxic gain-of-function versus overall loss-of-function), we generated LITAF KO (knock out) cells from control as well as T115N and L125P patient fibroblasts using CRISPR/Cas9. Cells were transduced with Cas9 and selected, before being transduced with gRNA against regions of LITAF conserved between WT and mutant LITAF and further selected. Mixed cell populations were generated and used for experiments because of the limited life spans of primary fibroblasts and our inability to grow single cell clonal lines to sufficient cell numbers. Western blotting confirmed the loss of LITAF expression in all three lines (Fig. 4a), and ~ 95% of cells were determined to be negative for any LITAF signal by confocal microscopy (Fig. 4b). Loss of LITAF from control fibroblasts induced phenotypes similar to those previously observed in T115N and L125P patient fibroblasts (Fig. 4c). Furthermore, the knockout of mutated LITAF from patient fibroblasts did not rescue the observed EM phenotype, and swollen compartments persisted in these cells (data for LITAF KO in T115N patient cells shown in Fig. 4c). Taken together, these data support the hypothesis that expression of LITAF harbouring CMT1C-associated pathogenic mutations cause an overall loss of function phenotype, which in isolation could be explained either by haploinsufficiency or by a dominant negative mechanism, mimicking the results seen following depletion of the wild type protein.

**Fig. 5:**
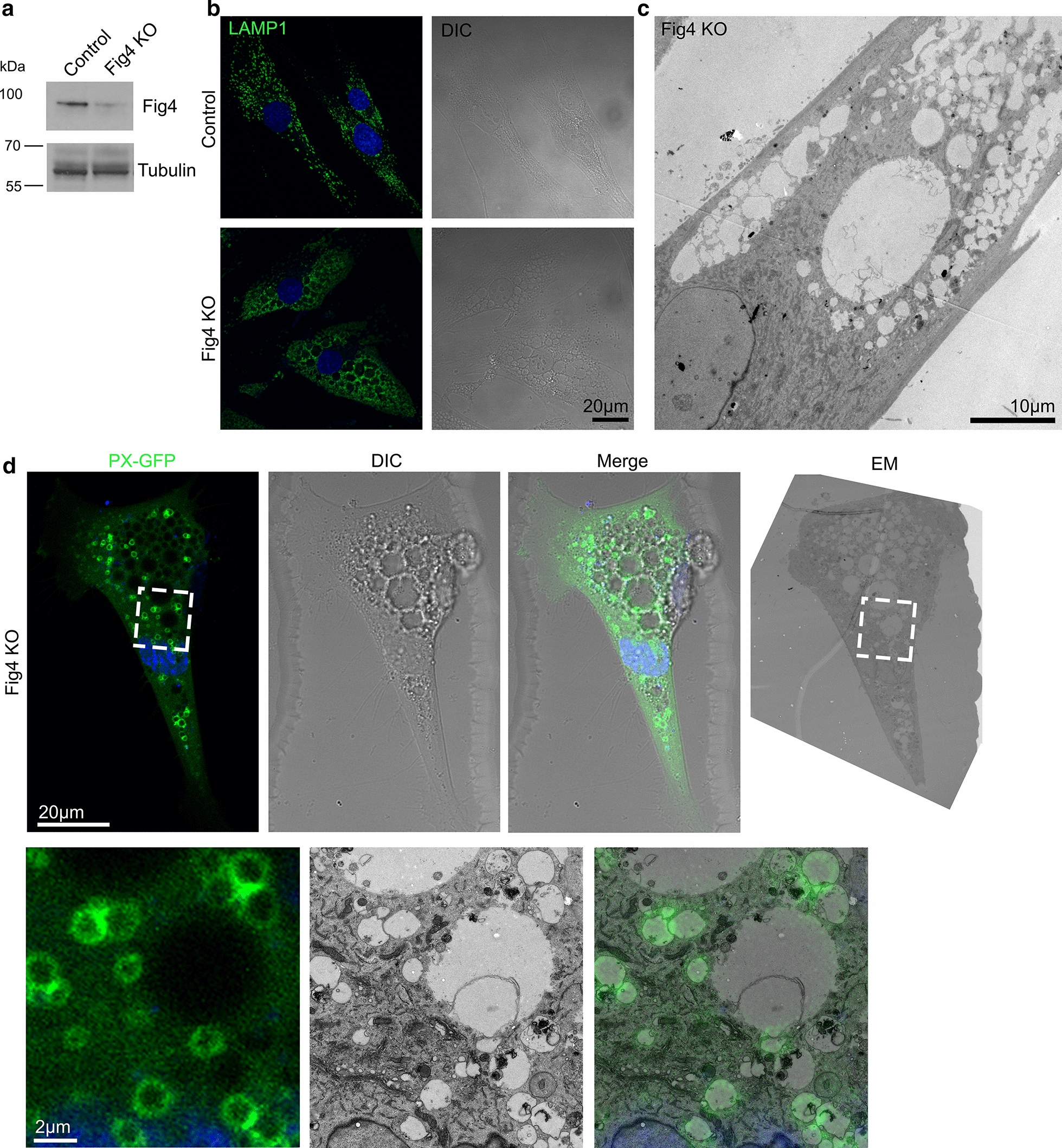
FIG4 KO fibroblasts display exaggerated swollen compartments. **a** Control fibroblasts were depleted for FIG4 by CRISPR/Cas9 and mixed populations were grown. Western blotting confirms loss of FIG4 protein. **b** FIG4 KO fibroblasts display swollen LAMP1 positive compartments that can also be seen by Differential Interference Contrast (DIC). **c** FIG4 KO fibroblasts contain vacuolar compartments visualised by conventional electron microscopy. **d** Correlative Light and Electron Microscopy (CLEM) of FIG4 KO fibroblasts transfected with the PtdIns(3)P probe, PX-GFP. GFP fluorescence displays overlap with smaller endocytic organelles, but not large compartments

As described above, FIG4 is a component of the PIKfyve complex and mutations in *FIG4* cause CMT4J. Disease-causing mutations in *FIG4,* depletion of FIG4 protein or inhibition of PIKfyve have all been reported to result in a morphological phenotype, with vacuolated late endosome and lysosome compartments [1, 2, 16, 44] and similarities to the phenotype we observed in the CMT1C patients’ fibroblasts containing mutant LITAF. To investigate this similarity further, we used a CRISPR/Cas9 protocol to generate FIG4 KO cells from control fibroblasts, the loss of protein in mixed cell populations being confirmed by western blotting (Fig. 5a). Using confocal immunofluorescence microscopy, we observed that FIG4 KO fibroblasts displayed enlarged, vacuolar LAMP1-positive compartments, often much larger than we had previously seen in the CMT1C patients’ cells (Fig. 5b). At the ultrastructural level, FIG4 KO fibroblasts displayed small vacuoles, as previously seen in CMT1C and LITAF KO fibroblasts, but additionally often displayed very large (over 10 μm) vacuoles (Fig. 5c). These vacuoles were largely devoid of any obvious luminal content and could also be seen with DIC (differential interference contrast) microscopy (Fig. 5d). As we had previously found the vacuoles observed in CMT1C cells to be PtdIns3P-rich, we wanted to determine whether the vacuoles generated following FIG4 KO were similarly rich in PtdIns(3)P, so we performed CLEM on FIG4 KO cells transiently expressing PX-GFP (Fig. 5d). Surprisingly, only the smaller vacuoles and not the larger vacuoles appeared to be labelled with PX-GFP. This suggests that the very large vacuoles present following FIG4 KO represent additional disruption to the endolysosomal system, rather than an exaggerated form of disruption as observed following LITAF mutation or knock out. However, it is also feasible that the enlarged surface area of these very big vacuoles may dilute the signal from PX-GFP to undetectable levels (Fig. 5d).

**Fig. 6:**
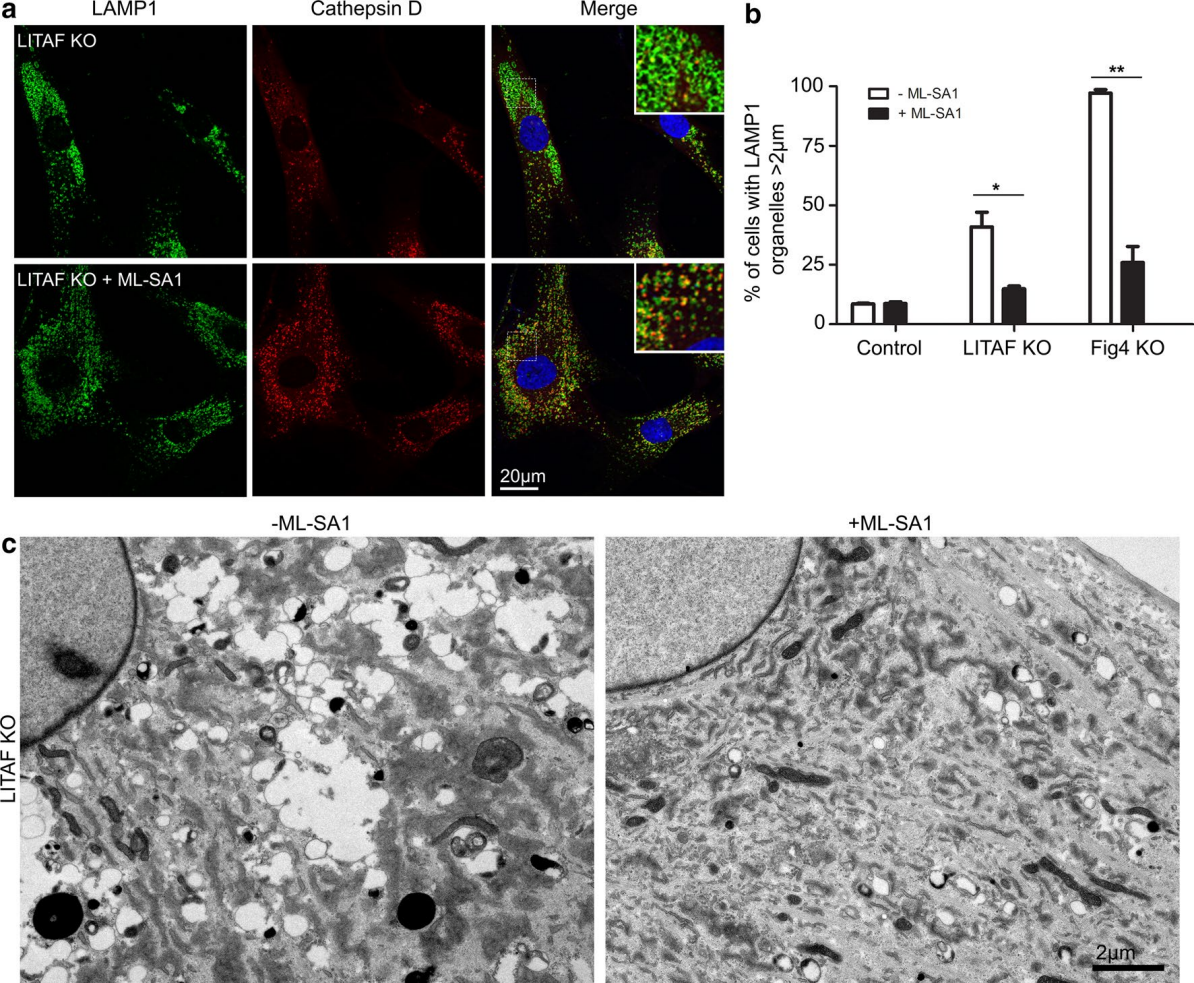
Enlarged endocytic compartments present in LITAF KO or FIG4 KO fibroblasts can be rescued by incubation of cells with the mucolipin activator, ML-SA1. **a** Immunofluorescence of LITAF KO fibroblasts display enlarged lysosomes, shown by LAMP1 fluorescence that can be reduced by incubation with the TRPML1 agonist, ML-SA1 (40 μM, 36 h). **b** Quantification of control, LITAF KO or Fig. 4 KO fibroblasts with LAMP1 organelles larger than 2um, with and without incubation with ML-SA1 (40 μM, 36 h). 3 independent experiments were performed, and from each experiment at least 20 cells were quantified. Data shown represents mean and S.E.M. Two-tailed, unpaired T-tests were used to analyse the statistical differences −/+ ML-SA1. *p < 0.05, **p < 0.01, ***p < 0.001. Total number of cells analysed; L261−ML-SA1, 106; L261+ML-SA1, 102; LITAF KO−ML-SA1, 96; LITAF KO+ML-SA1, 98; FIG4 KO–ML-SA1, 79; FIG4 KO + ML-SA1, 83). *P* values (L261; 0.8519, LITAFKO; 0.04676, FIG4 KO; 0.006978). **c** Conventional electron microscopy of LITAF KO fibroblasts without and with incubation with the TRPM1 agonist, ML-SA1 (40 μM, 36 h)

It has previously been shown that increased lysosome size observed in mouse FIG4 KO fibroblasts can be reduced by incubation with ML-SA1, a small molecule synthetic activator of the lysosomal cation channel TRPML1 [46]. This is consistent with FIG4′s normal function as part of the PIKfyve complex in synthesising PtdIns(3,5)P_2_, a natural activator of TRPML1. To see if ML-SA1 might also rescue the vacuolation phenotype in our human fibroblasts, we incubated control, LITAF KO and FIG4 KO fibroblasts with or without ML-SA1 (40 μM) for 36 h, immunolabelled LAMP1 and cathepsin D, then analysed late endosome/lysosome size by confocal microscopy (Fig. 6a, Additional file 4: Fig. 4). The diameter of LAMP1 positive organelles was measured and the percentage of cells with LAMP1 organelles over 2 μm calculated (Fig. 6b). Incubation of control fibroblasts with ML-SA1 did not alter the proportion of cells with LAMP1 organelles over 2 μm (mock, 8.53 ± 0.38%; + ML-SA1, 8.70 ± 0.74%). LITAF KO fibroblasts showed an increased proportion of cells with enlarged LAMP1 compartments (40.97 ± 6.14%), that was reduced following treatment with ML-SA1 (14.89 ± 1.17%). Consistent with the published data on mouse KO fibroblasts, the majority of human FIG4 KO fibroblasts displayed enlarged LAMP1 positive organelles (97.22 ± 1.39%), but the proportion of cells with enlarged organelles was dramatically reduced following ML-SA1 treatment (25.98 ± 6.76%). By EM, we observed a dramatic reduction in the appearance of vacuolated compartments in LITAF KO fibroblasts, FIG4 KO fibroblasts, and also CMT1C fibroblasts with the T115N LITAF mutation, after the incubation with ML-SA1 (Fig. 6c, Additional file 5: Fig. 5).

## Discussion

The development of effective treatments for inherited neurological disorders such as CMT remains an elusive goal. Despite rapid advances in the identification of mutations in disease-associated genes, these impressive discoveries have yet to be translated into proven therapies. This is particularly pertinent in CMT, where disease-associated mutations in more than 100 genes have now been described, leading us to question whether treatments for CMT will be subtype specific, or if therapeutic targets common to more than one-subtype can be identified. These efforts will ultimately depend on establishing the function and pathological consequences of CMT-associated mutations at the subcellular and cellular levels.

Our observations that pathological CMT-associated mutations in *LITAF* resulted in enlargement and vacuolation of late endosomal/lysosomal compartments, without any obvious effect on the morphology of compartments labelled with markers of early endosomes, are consistent with previous data showing that LITAF associates with endosomal membranes. However, the precise mechanism by which mutations in *LITAF* cause an autosomal dominant disease has been the subject of debate. That is, does the expression of LITAF harbouring CMT1C-associated pathogenic mutations lead to a toxic ‘gain of function,’ or instead results in loss of function, either by haploinsufficiency or by a ‘dominant-negative’ mechanism? The answer to this question has crucial implications for the development of therapeutic strategies for this subtype of demyelinating CMT.

Our data argue strongly that CMT1C is the result of overall loss of function of LITAF in vivo*,* rather than a toxic ‘gain of function.’ This is supported by the fact that we observed the same enlargement and vacuolation of endolysosomes in patient fibroblasts taken from CMT1C patients and in fibroblasts depleted of the LITAF protein, and consistent with the work of Zhu et al*.* who reported endolysosomal abnormalities in primary embryonic fibroblasts taken from mice either depleted of or expressing wild type and CMT1C-mutated Litaf protein at endogenous levels [45]. But is this loss of function due to haploinsufficiency (whereupon the expression of one wild-type allele is insufficient to maintain normal endolysosomes) or does LITAF protein harbouring CMT1C pathogenic mutations act in a ‘dominant-negative’ fashion (somehow impacting on the product of the wild-type allele leading to overall loss of function)? To address this, and arguing against haploinsufficiency, we can turn to the mice generated by Lee et al*.* where the two endogenous wild type *Litaf* alleles could not prevent the development of a demyelinating peripheral neuropathy in the presence of overexpressed exogenous LITAF harbouring the pathogenic W116G CMT1C-associated mutation [22]. Nevertheless, why the LITAF knock-out mice described by Somandin et al*.* were relatively unaffected is currently unclear, although the ultrastructure of any knock-out cells was not described in their study. This apparent physiological compensation could be explained by mechanisms such as the upregulation of other proteins at an early stage, possibly including the only other LITAF domain-containing protein encoded in the human genome, CDIP1 (cell death involved p53 target 1) or that the progressive peripheral nerve degeneration that characterises CMT in humans has yet to manifest in 18 month old mice [40]. Taken together, we therefore believe that our observations support a ‘dominant negative’ pathomechanisms that leads to disease in CMT1C, possibly mediated by the sequestering via oligomerisation of wild type LITAF by the mutant LITAF protein [14, 21] leading to mislocalisation, aggregation and overall loss of function in vivo [12, 21].

Disrupted and swollen endocytic compartments have previously been described in a different subtype of CMT, CMT4J, associated with loss of function of the lipid phosphatase, FIG4. Given the apparent morphological similarities between fibroblast taken from CMT1C patients and fibroblasts depleted of FIG4, we next focused on compartments of the late endocytic pathway. These compartments comprise late endosomes, endolysosomes and lysosomes, which are interactive, dynamic and play multiple functional roles such as macromolecule degradation, nutrient sensing, signalling to the cell nucleus, and plasma membrane repair [25, 36]. Late endosomes are multivesicular bodies formed by a maturation process from early endosomes [15] and deliver endocytosed macromolecules for degradation by lysosomal hydrolases through kissing and fusion with lysosomes. This process eventually results in the formation of endolysosomes, which are the principal sites of acid hydrolase activity and from which re-usable, terminal lysosomes are re-formed [4]. A key regulator of the dynamics of late endosomal/lysosomal compartments is the phosphoinositide, PtdIns(3,5)P_2_. Reduction of PtdIns(3,5)P_2_ synthesis by depletion or pharmacological inhibition of the lipid kinase PIKfyve leads to the disruption of homeostasis and the resulting vacuolation of late endosomal/lysosomal compartments due to an imbalance of lysosome fusion with endosomes and the reformation of lysosomes [2, 7, 9]. Both processes are mediated by the release of lysosomal Ca^2+^ via the TRPML1 channel, which is activated by PtdIns(3,5)P_2_ [8]. However, lysosome re-formation, which requires the presence of TRPML1, appears to be disproportionately inhibited by the absence of PtdIns(3,5)P_2_, possibly because lysosomal Ca^2+^ released into the cytosol is required for the extension and/or excision of the membrane bridges connecting endolysosomes to nascent re-forming lysosomes [27]. In addition to the vacuolation of late endocytic compartments due to inhibition of PIKfyve, mutations in *FIG4* or *VAC14*, encoding other components of the PIKfyve complex, also cause vacuolation of late endosomal/lysosomal compartments. Although FIG4 is a lipid phosphatase which can act on PtdIns(3,5)P_2_, its functional role in the PIKfyve complex appears to be phosphatase-independent and has been proposed to act alongside VAC14 in a scaffolding capacity to enable PIKfyve activity [1, 6, 23]. The role of Ca^2+^ release via TRPML1 can be seen in FIG4 KO fibroblasts where partial rescue of the vacuolation phenotype can be achieved in these cells by incubation with the small molecule synthetic activator of TRPML1, ML-SA1 ( [46] and see Results section). Furthermore, and consistent with disruption of PIKfyve activity, fibroblasts taken from a patient with a biallelic loss-of-function variant in VAC14 were recently reported to contain numerous swollen cytoplasmic vacuoles, a phenotype that was also partially rescued by ML-SA1 [24]. Although a similar rescue of vacuolation with ML-SA1 was not reported following pharmacological inhibition of PIKfyve in HeLa cells [2], this apparently negative finding could be explained by the much shorter incubation time used, 2 h in HeLa cells compared to 24 h or longer with the FIG4 KO and VAC14 patient cells [24, 46]. Whether this implies an effect beyond simply modulating the amount of fusion and fission of lysosomes is unclear. Certainly, release of lysosomal Ca^2+^ through TRPML1 can have other effects, not least in stimulating dephosphorylation of transcription factor EB, resulting in its entry into the nucleus and upregulating genes required for lysosome biogenesis [26].

Our observation that the enlargement and vacuolation of late endosomal/lysosomal compartments in CMT1C and LITAF KO fibroblasts can also be partially reversed with ML-SA1 strongly suggests that LITAF functions on the same endolysosomal pathway as FIG4. LITAF is an endosomal membrane protein but whether it interacts with the machinery of PtdIns(3,5)P_2_ production or has an independent effect on TRPML1 is presently unknown.

CMT1C manifests clinically as a demyelinating sensorimotor neuropathy where Schwann cell dysfunction is presumed to lead to peripheral nerve demyelination. Contrary to other subtypes of demyelinating CMT where the mutated proteins appear to play specific and specialised roles in Schwann cell function (e.g. SH3TC2, MPZ, PMP22, EGR2), LITAF is widely expressed across many cell types and tissues. Therefore, in contrast to other tissues and cells which have capacity to functionally compensate, Schwann cells might be exquisitively sensitive to cumulative endocytic disruption over time, leading to the eventual inability to maintain the highly complex state of myelination. This notion is supported by the number of genes found to be associated with CMT that have known or predicted roles in endocytic membrane traffic. Furthermore, while CMT1C is characterised by an isolated progressive demyelinating peripheral neuropathy, CMT4J is recognised as a much more severe autosomal recessive form of the disease. It is therefore tempting to speculate that these differential clinical observations are related to the very large vaculoes (over 10 μm) found in FIG4 KO fibroblasts but not in fibroblasts from CMT1C patients or in fibroblasts depleted of wild type LITAF.

Our investigations have been conducted on fibroblasts, but it is the functional effects of LITAF and FIG4 mutations in Schwann cells that are likely to help explain the underlying pathomechanism of these inherited demyelinating peripheral neuropathies. This raises the question of which specialised pathways in Schwann cells might be vulnerable as a result of the enlargement and vacuolation of late endosomal/lysosomal compartments. Interestingly, although distinct differences exist between the formation and maintenance of myelin in the central and peripheral nervous systems, the loss of FIG4 in mouse oligodendrocytes (the myelin-forming glia of the central nervous system) leads to the accumulation of specific strucutral myelin proteins such as MAG (Myelin Associated Glycoprotein) within perinuclear LAMP-1 positive vesicles [28]. Similarly, the maintenance of peripheral nerve myelination and the structural integrity of specialised regions such as the nodes of Ranvier and adjacent paranodes rely on the trafficking and recycling of specialised membrane receptors in Schwann cells. For example, the interactions between Schwann cells and axons are mediated in part by various cellular adhesion molecules such as integrins; a family of transmembrane proteins that facilitate interactions between cells and the extracellular matrix, along with mediating specific cell–cell interactions. Integrins are heterodimers formed by stable non-covalently associated α and β subunits. 18 α and 8 β subunits form 24 distinct integrin heterodimers, although integrins of the β1 family predominate in the peripheral nervous system and loss of integrin function impairs the abilty of Schwann cells to interact properly with axons [11, 29]. β1 integrins recycle to the plasma membrane from endocytic compartments, including late endosomes [31] and disruption to the endocytic pathway reduces β1 integrin recycling to the plasma membrane [33]. We therefore hypothesise that endosomal dysfunction might alter the trafficking of key membrane proteins such as β1 integrin in a number of demyelinating CMTs of different subtypes (such as CMT1C and CMT4J), and that TRPML1 activators may be an attractive starting point for the development of drugs that may stabilise and also reverse the clinical phenotypes associated with more than one subtype of this currently untreatable group of inherited neuromuscular disorders.

## Supplementary information

Additional file 1: Fig. 1 Comparison of markers of the endomembrane system between control and LITAF mutant patient fibroblasts. Control and LITAF mutant fibroblasts (L125P, T115N) displayed no consistent differences in staining for several markers of endocytic compartments, autophagosomes or the Golgi, as shown by confocal immunofluorescence microscopy using antibodies against APPL1, EEA1, CD63, LBPA, ubiquitin, LC3B, CIMPR and TGN46.

Additional file 2: Fig. 2 Full-length blots. Full-length blots from Figs. 1d and 4a. Cropped areas are highlighted and labelled.

Additional file 3: Fig. 3 Swollen compartments are accessible to endocytic tracers. Further examples of Fig. 2b. Control or LITAF mutant fibroblasts were incubated with fluid-phase HRP for 4 h before being fixed. A DAB reaction was performed on cells before being processed for conventional electron microscopy.

Additional file 4: Fig. 4 ML-SA1 rescues enlarged LAMP1 positive compartments from LITAF KO and FIG4 KO fibroblasts. Immunofluorescence showing LAMP1 and Cathepsin D labelling in control, LITAF KO and FIG4 KO fibroblasts without and with treatment with ML-SA1 (40μM, 36 h).

Additional file 5: Fig. 5 ML-SA1 reduces the frequency of vacuolar endocytic compartments from T115N LITAF patient fibroblasts, and FIG4KO fibroblasts. Conventional electron microscopy of T115N fibroblasts (a) and FIG4 KO fibroblasts (b) without and with treatment with ML-SA1 (40μM, 36 h).

## Data Availability

The data that support the findings of this study are available from the corresponding author, upon reasonable request.

## Abbreviations

APPL1: Adaptor protein, phosphotyrosine interacting with PH domain and leucine zipper 1
CDIP1: Cell death involved p53 target 1
ciMPR: Cation independent mannose 6-phosphate receptor
CLEM: Correlative light and electron microscopy
CMT: Charcot–Marie–Tooth
EEA1: Early endosome antigen 1
EM: Electron microscopy
ESCRT: Endocytic sorting complex required for transport
FIG4: Fact or -induced gene 4
LAMP1: Lysosomal-associate membrane protein 1
LBPA: Lysosbisphosphatidic acid
LC3: Microtubule-associated protein 1 light chain 3
LITAF: Lipopolysaccharide-induced TNF factor
MTMR: Myotubularin-related protein
PI: Phosphatidylinositol
PIKfyve: Phosphoinositide kinasefyve-type
PtdIns(3)P: Phosphatidylinositol 3-phosphate
PtdIns(3,5)P_2_: Phosphatidylinositol 3,5-bisphosphate
SIMPLE: Small integral membrane protein of the lysosome/late endosome
TGN46: Trans-Golgi network 46
TRPML1: Transient receptor potential mucolipin1
TSG101: Tumor susceptibility gene 101

## References

[1] Bharadwaj R, Cunningham KM, Zhang K, Lloyd TE (2016) FIG4 regulates lysosome membrane homeostasis independent of phosphatase function. Hum Mol Genet 25:681–692. 10.1093/HMG/DDV505 26662798 10.1093/hmg/ddv505 PMC4743688

[2] Bissig C, Hurbain I, Raposo G, van Niel G (2017) PIKfyve activity regulates reformation of terminal storage lysosomes from endolysosomes. Traffic 18:747–757. 10.1111/tra.12525 28857423 10.1111/tra.12525

[3] Brennan KM, Bai Y, Shy ME (2015) Demyelinating CMT-what’s known, what’s new and what’s in store? Neurosci Lett 596:14–26 25625223 10.1016/j.neulet.2015.01.059

[4] Bright NA, Reaves BJ, Mullock BM, Luzio JP (1997) Dense core lysosomes can fuse with late endosomes and are re-formed from the resultant hybrid organelles. J Cell Sci 110:2027–2040 9378754 10.1242/jcs.110.17.2027

[5] Bucci C, Bakke O, Progida C (2012) Charcot-Marie-Tooth disease and intracellular traffic. Prog Neurobiol 99:191–225 22465036 10.1016/j.pneurobio.2012.03.003 PMC3514635

[6] Chow CY, Zhang Y, Dowling JJ, Jin N, Adamska M, Shiga K, Szigeti K, Shy ME, Li J, Zhang X, Lupski JR, Weisman LS, Meisler MH (2007) Mutation of FIG4 causes neurodegeneration in the pale tremor mouse and patients with CMT4J. Nature 448:68–72. 10.1038/nature05876 17572665 10.1038/nature05876 PMC2271033

[7] Choy CH, Saffi G, Gray MA, Wallace C, Dayam RM, Ou ZYA, Lenk G, Puertollano R, Watkins SC, Botelho RJ (2018) Lysosome enlargement during inhibition of the lipid kinase PIKfyve proceeds through lysosome coalescence. J Cell Sci. 10.1242/JCS.213587 29661845 10.1242/jcs.213587 PMC6031331

[8] Dong XP, Shen D, Wang X, Dawson T, Li X, Zhang Q, Cheng X, Zhang Y, Weisman LS, Delling M, Xu H (2010) PI(3,5)P2 controls membrane trafficking by direct activation of mucolipin Ca2+ release channels in the endolysosome. Nat Commun. 10.1038/ncomms1037 20802798 10.1038/ncomms1037 PMC2928581

[9] Dove SK, Dong K, Kobayashi T, Williams FK, Michell RH (2009) Phosphatidylinositol 3,5-bisphosphate and Fab1p/PIKfyve underPPIn endo-lysosome function. Biochemistry J 419:1–13 10.1042/BJ20081950 19272020

[10] Eaton HE, Desrochers G, Drory SB, Metcalf J, Angers A, Brunetti CR (2011) SIMPLE/LITAF expression induces the translocation of the ubiquitin ligase itch towards the lysosomal compartments. PLoS ONE 6:e16873. 10.1371/journal.pone.0016873 21326863 10.1371/journal.pone.0016873 PMC3033906

[11] Feltri ML, Porta DG, Previtali SC, Nodari A, Migliavacca B, Cassetti A, Littlewood-Evans A, Reichardt LF, Messing A, Quattrini A, Mueller U, Wrabetz L (2002) Conditional disruption of β1 integrin in Schwann cells impedes interactions with axons. J Cell Biol 156:199–209. 10.1083/jcb.200109021 11777940 10.1083/jcb.200109021 PMC2173589

[12] Ferreira Lacerda A, Hartjes E, Brunetti CR (2014) LITAF mutations associated with charcot-marie-tooth disease 1C show mislocalization from the late endosome/lysosome to the mitochondria. PLoS ONE 9:e103454. 10.1371/journal.pone.0103454 25058650 10.1371/journal.pone.0103454 PMC4110028

[13] Gruenberg J (2020) Life in the lumen: the multivesicular endosome. Traffic 21:76–93. 10.1111/tra.12715 31854087 10.1111/tra.12715 PMC7004041

[14] Ho AK, Wagstaff JL, Manna PT, Wartosch L, Qamar S, Garman EF, Freund SMV, Roberts RC (2016) The topology, structure and PE interaction of LITAF underpin a Charcot–Marie–Tooth disease type 1C. BMC Biol 14:109. 10.1186/s12915-016-0332-8 27927196 10.1186/s12915-016-0332-8 PMC5142333

[15] Huotari J, Helenius A (2011) Endosome maturation. EMBO J 30:3481–3500 21878991 10.1038/emboj.2011.286 PMC3181477

[16] Katona I, Zhang X, Bai Y, Shy ME, Guo J, Yan Q, Hatfield J, Kupsky WJ, Li J (2011) Distinct pathogenic processes between Fig4-deficient motor and sensory neurons. Eur J Neurosci 33:1401–1410. 10.1111/j.1460-9568.2011.07651.x 21410794 10.1111/j.1460-9568.2011.07651.x

[17] Laporte J, Bedez F, Bolino A, Mandel JL (2003) Myotubularins, a large disease-associated family of cooperating catalytically active and inactive phosphoinositides phosphatases. Hum Mol Genet 12:R285–R292 12925573 10.1093/hmg/ddg273

[18] Lartigue de J, Polson H, Feldman M, Shokat K, Tooze SA, Urbé S, Clague MJ (2009) PIKfyve regulation of endosome-linked pathways. Traffic 10:883–893. 10.1111/j.1600-0854.2009.00915.x 19582903 10.1111/j.1600-0854.2009.00915.x PMC2723830

[19] Laurá M, Pipis M, Rossor AM, Reilly MM (2019) Charcot-marie-Tooth disease and related disorders: an evolving landscape. Curr Opin Neurol 32:641–650 31343428 10.1097/WCO.0000000000000735

[20] Lee SM, Chin LS, Li L (2012) Charcot-marie-tooth disease-linked protein SIMPLE functions with the ESCRT machinery in endosomal trafficking. J Cell Biol 199:799–816. 10.1083/jcb.201204137 23166352 10.1083/jcb.201204137 PMC3514783

[21] Lee SM, Olzmann JA, Chin LS, Li L (2011) Mutations associated with Charcot–Marie–Tooth disease cause SIMPLE protein mislocalization and degradation by the proteasome and aggresome-autophagy pathways. J Cell Sci 124:3319–3331. 10.1242/jcs.087114 21896645 10.1242/jcs.087114 PMC3178453

[22] Lee SM, Sha D, Mohammed AA, Asress S, Glass JD, Chin LS, Li L (2013) Motor and sensory neuropathy due to myelin infolding and paranodal damage in a transgenic mouse model of charcot-marie-tooth disease type 1c. Hum Mol Genet 22:1755–1770. 10.1093/hmg/ddt022 23359569 10.1093/hmg/ddt022 PMC3613163

[23] Lenk GM, Frei CM, Miller AC, Wallen RC, Mironova YA, Giger RJ, Meisler MH (2016) Rescue of neurodegeneration in the Fig4 null mouse by a catalytically inactive FIG4 transgene. Hum Mol Genet 25:340–347. 10.1093/hmg/ddv480 26604144 10.1093/hmg/ddv480 PMC4706117

[24] Lines MA, Ito Y, Kernohan KD, Mears W, Hurteau-Miller J, Venkateswaran S, Ward L, Khatchadourian K, McClintock J, Bhola P, Campeau PM, Boycott KM, Michaud J, Van Kuilenburg ABP, Ferdinandusse S, Dyment DA (2017) Yunis-Varón syndrome caused by biallelic VAC14 mutations. Eur J Hum Genet 25:1049–1054. 10.1038/ejhg.2017.99 28635952 10.1038/ejhg.2017.99 PMC5558182

[25] Luzio JP, Pryor PR, Bright NA (2007) Lysosomes: fusion and function. Nat Rev Mol Cell Biol 8:622–632 17637737 10.1038/nrm2217

[26] Medina DL, Di Paola S, Peluso I, Armani A, De Stefani D, Venditti R, Montefusco S, Scotto-Rosato A, Prezioso C, Forrester A, Settembre C, Wang W, Gao Q, Xu H, Sandri M, Rizzuto R, De Matteis MA, Ballabio A (2015) Lysosomal calcium signalling regulates autophagy through calcineurin and TFEB. Nat Cell Biol 17:288–299. 10.1038/ncb3114 25720963 10.1038/ncb3114 PMC4801004

[27] Miller A, Schafer J, Upchurch C, Spooner E, Huynh J, Hernandez S, Mclaughlin B, Oden L, Fares H (2015) Mucolipidosis type IV protein TRPML1-dependent lysosome formation. Traffic 16:284–297. 10.1111/tra.12249 25491304 10.1111/tra.12249

[28] Mironova YA, Lenk GM, Lin JP, Lee SJ, Twiss JL, Vaccari I, Bolino A, Havton LA, Min SH, Abrams CS, Shrager P, Meisler MH, Giger RJ (2016) PI(3,5)P2 biosynthesis regulates oligodendrocyte differentiation by intrinsic and extrinsic mechanisms. Elife 5:1–29. 10.7554/eLife.13023 10.7554/eLife.13023 PMC4889328 27008179

[29] Ness JK, Snyder KM, Tapinos N (2013) Lck tyrosine kinase mediates β1-integrin signalling to regulate Schwann cell migration and myelination. Nat Commun 4:1912. 10.1038/ncomms2928 23715271 10.1038/ncomms2928 PMC3674276

[30] Nicholson G, Lenk GM, Reddel SW, Grant AE, Towne CF, Ferguson CJ, Simpson E, Scheuerle A, Yasick M, Hoffman S, Blouin R, Brandt C, Coppola G, Biesecker LG, Batish SD, Meisler MH (2011) Distinctive genetic and clinical features of CMT4J: a severe neuropathy caused by mutations in the PI(3,5)P2 phosphatase FIG4. Brain 134:1959–1971. 10.1093/brain/awr148 21705420 10.1093/brain/awr148 PMC3122378

[31] Powelka AM, Sun J, Li J, Gao M, Shaw LM, Sonnenberg A, Hsu VW (2004) Stimulation-dependent recycling of integrin β1 regulated by ARF6 and Rab11. Traffic 5:20–36 14675422 10.1111/j.1600-0854.2004.00150.x

[32] Qin W, Wunderley L, Barrett AL, High S, Woodman PG (2016) The charcot marie tooth disease protein LITAF is a zinc-binding monotopic membrane protein. Biochemistry J 473:3965–3978. 10.1042/BCJ20160657 10.1042/BCJ20160657 PMC5095921 27582497

[33] Ratcliffe CDH, Sahgal P, Parachoniak CA, Ivaska J, Park M (2016) Regulation of cell migration and β1 integrin trafficking by the endosomal adaptor GGA3. Traffic 17:670–688. 10.1111/tra.12390 26935970 10.1111/tra.12390

[34] Rossor AM, Polke JM, Houlden H, Reilly MM (2013) Clinical implications of genetic advances in charcot-marie-tooth disease. Nat Rev Neurol 9:562–571. 10.1038/nrneurol.2013.179 24018473 10.1038/nrneurol.2013.179

[35] Schiza N, Georgiou E, Kagiava A, Médard JJ, Richter J, Tryfonos C, Sargiannidou I, Heslegrave AJ, Rossor AM, Zetterberg H, Reilly MM, Christodoulou C, Chrast R, Kleopa KA (2019) Gene replacement therapy in a model of Charcot–Marie–Tooth 4C neuropathy. Brain 142:1227–1241. 10.1093/brain/awz064 30907403 10.1093/brain/awz064 PMC6487329

[36] Settembre C, Fraldi A, Medina DL, Ballabio A (2013) Signals from the lysosome: a control centre for cellular clearance and energy metabolism. Nat Rev Mol Cell Biol 14:283–296 23609508 10.1038/nrm3565 PMC4387238

[37] Shirk AJ, Anderson SK, Hashemi SH, Chance PF, Bennett CL (2005) SIMPLE interacts with NEDD4 and TSG101: evidence for a role in lysosomal sorting and implications for Charcot–Marie–Tooth disease. J Neurosci Res 82:43–50. 10.1002/jnr.20628 16118794 10.1002/jnr.20628

[38] Slot JW, Geuze HJ (1985) A new method of preparing gold probes for multiple-labeling cytochemistry. Eur J Cell Biol 38:87–93 4029177

[39] Slot JW, Geuze HJ, Gigengack S, Lienhard GE, James DE (1991) Immuno-localization of the insulin regulatable glucose transporter in brown adipose tissue of the rat. J Cell Biol 113:123–135. 10.1083/jcb.113.1.123 2007617 10.1083/jcb.113.1.123 PMC2288909

[40] Somandin C, Gerber D, Pereira JA, Horn M, Suter U (2012) LITAF (SIMPLE) regulates Wallerian degeneration after injury but is not essential for peripheral nerve development and maintenance: implications for Charcot–Marie–Tooth disease. Glia 60:1518–1528. 10.1002/glia.22371 22729949 10.1002/glia.22371

[41] Stojkovic T (2016) Hereditary neuropathies: an update. Rev Neurol (Paris) 172:775–778 27866730 10.1016/j.neurol.2016.06.007

[42] Vaccari I, Dina G, Tronchère H, Kaufman E, Chicanne G, Cerri F, Wrabetz L, Payrastre B, Quattrini A, Weisman LS, Meisler MH, Bolino A (2011) Genetic interaction between MTMR2 and FIG4 phospholipid phosphatases involved in Charcot–Marie–Tooth neuropathies. PLoS Genet 7:e1002319. 10.1371/journal.pgen.1002319 22028665 10.1371/journal.pgen.1002319 PMC3197679

[43] van Niel G, Charrin S, Simoes S, Romao M, Rochin L, Saftig P, Marks MS, Rubinstein E, Raposo G (2011) The tetraspanin CD63 regulates ESCRT-independent and -dependent endosomal sorting during melanogenesis. Dev Cell 21:708–721. 10.1016/j.devcel.2011.08.019 21962903 10.1016/j.devcel.2011.08.019 PMC3199340

[44] Zhang X, Chow CY, Sahenk Z, Shy ME, Meisler MH, Li J (2008) Mutation of FIG4 causes a rapidly progressive, asymmetric neuronal degeneration. Brain 131:1990–2001. 10.1093/brain/awn114 18556664 10.1093/brain/awn114 PMC2724900

[45] Zhu H, Guariglia S, Yu RYL, Li W, Brancho D, Peinado H, Lyden D, Salzer J, Bennett C, Chow CW (2013) Mutation of SIMPLE in Charcot–Marie–Tooth 1C alters production of exosomes. Mol Biol Cell 24:1619–1637. 10.1091/mbc.E12-07-0544 23576546 10.1091/mbc.E12-07-0544 PMC3667717

[46] Zou J, Hu B, Arpag S, Yan Q, Hamilton A, Zeng YS, Vanoye CG, Li J (2015) Reactivation of lysosomal Ca2+ efflux rescues abnormal lysosomal storage in FIG4-deficient cells. J Neurosci 35:6801–6812. 10.1523/JNEUROSCI.4442-14.2015 25926456 10.1523/JNEUROSCI.4442-14.2015 PMC4412898

